# A Case of Goldenhar Syndrome Associated with a New Retinal Presentation: Exudative Vitelliform Maculopathy

**DOI:** 10.1155/2015/626027

**Published:** 2015-05-03

**Authors:** Claudia Bruè, Cesare Mariotti, Silvia Celani, Ilaria Rossiello, Alfonso Giovannini

**Affiliations:** Ophthalmology, Department of Neuroscience, Polytechnic University of Marche, 60039 Ancona, Italy

## Abstract

Goldenhar syndrome is a rare clinical disturbance with a wide range of clinical manifestations. We report on a 6-year-old male with peculiar retinal presentation of Goldenhar syndrome. The patient was referred to Ophthalmology for central scotoma in the left eye, where visual acuity was 20/100. Fundus examination was unremarkable, except for yellowish material in the central macula. SD-OCT revealed interruption of the external limiting membrane and inner and outer segment junctions, with disorganized material in the vitelliform space and subretinal fluid. Six months later, fundus and SD-OCT examinations were unchanged without treatment, but visual acuity in the left eye had improved to 20/50. Five years later, he had similar clinical manifestations in the right eye. He was started on systemic steroids. After 15 days, his visual acuity improved to 20/20 and subretinal fluid and yellowish material in the vitelliform space disappeared. Goldenhar syndrome has variable presentation, including vitelliform maculopathy.

## 1. Introduction

Goldenhar syndrome is also known as hemifacial microsomia and oculo-auriculo-vertebral dysplasia, and it derives from aberrant development of the first and second branchial arches [1]. It has a prevalence from 1 : 3500 to 1 : 7000 live births, with a male to female ratio of 3 : 2. Goldenhar syndrome shows a range of clinical manifestations, including microtia, hemifacial microsomia, preauricular skin tags, vertebral malformations, and epibulbar dermoids [2, 3]. Further skeletal abnormalities and ocular, renal, and cardiac abnormalities have been described [3]. We believe this is the first case report to describe Goldenhar syndrome in association with a vitelliform-like maculopathy.

## 2. Case Presentation

A 6-year-old Caucasian male had sudden blurring in the central visual field of the left eye. The child was born after a normal full-term delivery, with no history of maternal illness during pregnancy. He had facial asymmetry, hypoplastic maxilla, dysmorphic ear, and diminished hearing and had undergone surgery for auricular and maxillary abnormalities some months earlier. The child had a VMD2 gene mutation and a positive family history for vitelliform maculopathy.

**Figure 1:**
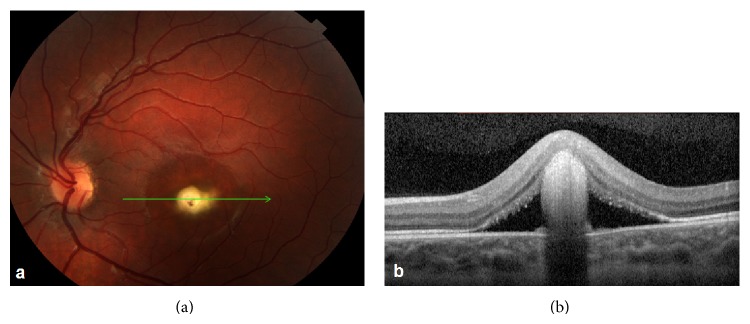
(a) Ophthalmoscopy of the right eye demonstrates round yellow material juxtafoveally, surrounded by subretinal fluid. (b) SD-OCT shows loss of foveal contour, interruption of the external limiting membrane, and the IS/OS junction, with disorganized material in the “vitelliform space” and subretinal fluid.

His visual acuity was 20/100 in the left eye. Anterior segment examination was unremarkable in each eye. On dilated fundus examination, the foveal reflex was reduced, with a round yellowish juxtafoveal lesion (Figure 1(a)). Spectral-domain optical coherence tomography (SD-OCT; Topcon America, Paramus, NJ) revealed loss of foveal contour and a small interruption in the external limiting membrane and the inner and outer segment (IS/OS) junctions, with disorganized material in the “vitelliform space” and subretinal fluid (Figure 1(b)). No therapy was given at that time. After 6 months, visual acuity improved to 20/50 with a refraction of +1.75 D and with slight attenuation of the lesions on fundus examination.

**Figure 2:**
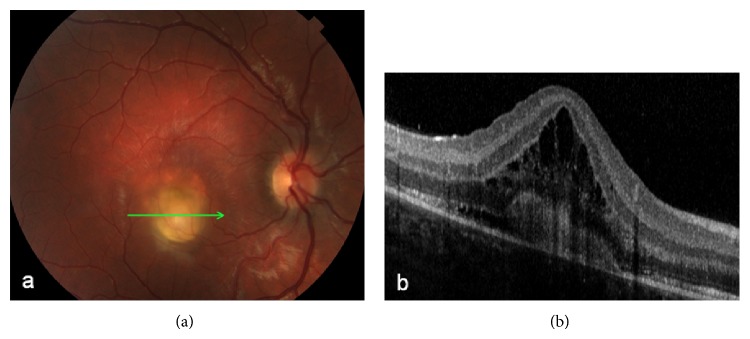
(a) The color fundus of the right eye shows a yellowish material at the center of the macula. (b) SD-OCT detects “vitelliform material” underlined by intraretinal cystic cavities and subretinal fluid.

**Figure 3:**
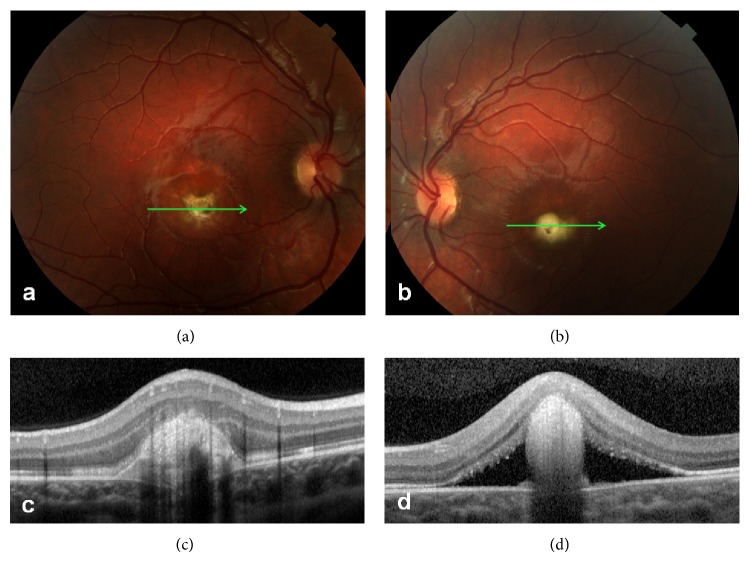
Fifteen days after high-dose steroid therapy. (a, b) Ophthalmoscopy demonstrates round yellow juxtafoveal material circled by pigment in the right eye (a) and yellowish foveal material surrounded by fluid in the left eye (b). (c, d) SD-OCT confirms hyper-reflective material in the vitelliform space and no fluid in the right eye (c), and smaller round hyper-reflective mass in the vitelliform space with adjacent subretinal fluid in the left eye (d).

After 5 years, he had sudden blurring of vision in the right eye, where visual acuity was 20/100. Fundus examination revealed yellowish material at the center of the macula (Figure 2(a)). SD-OCT again showed “vitelliform material” underlined by intraretinal cystic cavities and subretinal fluid (Figure 2(b)). High-dose steroid therapy was started (betamethasone, 1.5 mg/day). At 15 days of follow-up, visual acuity improved to 20/20 in the right eye and remained stable at 20/50 in the left eye. Fundus examination demonstrated round yellow juxtafoveal material surrounded by pigment in the right eye (Figure 3(a)), and yellowish foveal material surrounded by fluid in the left eye (Figure 3(b)). SD-OCT confirmed hyper-reflective material in the vitelliform space and no fluid in the right eye (Figure 3(c)), and a smaller round hyper-reflective mass in the vitelliform space with adjacent subretinal fluid in the left eye (Figure 3(d)).

## 3. Discussion

Goldenhar syndrome is an infrequent disorder of the first and second branchial arches [4]. It was first described by Goldenhar in 1952, as a triad of craniofacial microsomia, spinal anomalies, and ocular dermoid cysts, where ocular signs included dermoids and lipodermoids [5]. In 1967, Sugar described these as the most important ocular futures of Goldenhar syndrome, as bilateral in two-thirds of cases [6]. The dermoid is usually located in the lower outer quadrant and the lipodermoid in the upper outer quadrant. These lesions can cause amblyopia and strabismus, although with identification and early excision of the limbal dermoid lesions, amblyopia, and strabismus can be avoided. Consequently, this ameliorates the visual prognosis of Goldenhar patients.

Another frequent ocular lesion is unilateral coloboma of the upper lid. This form of dermoid or lipodermoid benign tumor has the epibulbar choristoma located on the inferotemporal or superotemporal part of the limbus, which represents the main ocular feature of Goldenhar syndrome (30%–60% of patients) [7]. Corneal and sclera infiltration by the tumors is rare, although this can lead to astigmatism. Correlated factors caused by eyelid mobility disorders, such as irritation and exposure keratitis, can induce central visual axis obscuration. The dermoid mass can permeate the cornea and thus provoke important astigmatism, with consequent amblyopia. Other ocular anomalies are rare but include microphthalmos, microcornea, anophthalmos, eyelid colobomas, iris and choroid colobomas, motility disorders, strabismus, blepharoptosis, palpebral fissure, iris atrophy, polar cataract, anomalous lacrimal drainage system, and retinal and optic nerve anomalies [8].

To the best of our knowledge, this is the first description of exudative vitelliform maculopathy associated with Goldenhar syndrome. As indicated by the clinical description and the facial features shown by the patient, this case gave specific indications for ocular symptoms and pathology beyond the more standard clinical descriptions of patients with Goldenhar syndrome. Thus, there appeared to have been some indications of vitelliform maculopathy as an associated ocular condition that would have allowed for the earlier diagnosis and treatment of this disorder.

There is no evidence of clear inheritance patterns for Goldenhar syndrome, with no chromosomal anomalies reported. Thus, Goldenhar syndrome appears to occur randomly, without apparent cause, although some family histories have suggested autosomal dominant or recessive inheritance. Some other studies hypothesized that this condition is caused by interactions of many genes, probably in combination with environmental factors, and multifactorial inheritance [9].

Vitelliform maculopathy (Best disease) is an autosomal dominant disorder with variable penetrance and is characterized by various depositions of yellowish material formed of lipofuscin in the retinal pigment epithelium and subretinal space [10]. The basic defect in vitelliform maculopathy is a mutated* Best1* gene, which codes for bestrophin, a Ca^2+^-sensitive Cl^−^-channel protein located on the basolateral membrane of retinal pigment epithelium cells. Abnormal chloride conductance caused by the mutated* Best1* gene can disturb fluid transport across the retinal pigment epithelium, which can then cause accumulation of debris between the retinal pigment epithelium and the photoreceptors, and between the retinal pigment epithelium and Bruch's membrane [10]. Our patient had a positive family history of Goldenhar syndrome, as his mother, an uncle, his grandmother, and his cousins were also affected. Therefore, it can be hypothesized that Goldenhar syndrome and vitelliform maculopathy share the same inheritance pattern anomaly.

In 1963, Braley and Spivey described beneficial effects of systemic steroids administered for over a year for patients affected by vitelliform maculopathy [10]. They defined a distinct rationale for systemic steroids, indicating that their use guaranteed good final visual acuity or at least a delay in severe visual loss. Also in our case, the therapeutic response to steroids suggests that these fundus disorders are caused by inflammatory, and probably immune-mediated, disorders. Rapid recovery of vision and resolution of subretinal exudation suggest that steroids stimulate proliferation of nonpigmented retinal pigment epithelium cells.

In conclusion, our case suggests multifactorial pathogenesis of Goldenhar syndrome and highlights the importance of knowledge of possible associations of vitelliform maculopathy with Goldenhar syndrome, to allow for early diagnosis and treatment of these disorders, and thus to prevent development of secondary amblyopia.

## References

[1] Sharma J. K., Pippal S. K., Raghuvanshi S. K., Shitij A. (2006). Goldenhar-Gorlin's syndrome: a case report. *Indian Journal of Otolaryngology and Head and Neck Surgery*.

[2] Martelli H., de Miranda R. T., Fernandes C. M. (2010). Goldenhar syndrome: clinical features with orofacial emphasis. *Journal of Applied Oral Science*.

[3] Rollnick B. R., Kaye C. I., Nagatoshi K., Hauck W., Martin A. O. (1986). Oculoauriculovertebral dysplasia and variants: phenotypic characteristics of 294 patients. *American Journal of Medical Genetics*.

[4] De Golovine S., Wu S., Hunter J. V., Sheater W. T. (2012). Goldenhar syndrome: a cause of secondary immunodeficiency?. *Allergy, Asthma & Clinical Immunology*.

[5] Goldenhar M. (1952). Associations malformatives de l'oeil et l'oreille, en particulier le syndrome dermoide épibulbaire-appendices auriculaires-fistula auris congenita et ses relations avec la dysostose mandibulo-faciale. *Journal of Human Genetics*.

[6] Sugar H. S. (1967). An unusual example of the oculo-auriculovertebral dysplasia syndrome of Goldenhar. *Journal of Pediatric Ophthalmology*.

[7] Aleksic S., Budzilovich G., Reuben R. (1975). Congenital trigeminal neuropathy in oculoauriculovertebral dysplasia-hemifacial microsomia (Goldenhar-Gorlin syndrome). *Journal of Neurology Neurosurgery and Psychiatry*.

[8] Bhallil S., Benatiya I., El Abdouni O., Mahjoubi B., Hicham T. (2010). Goldenhar syndrome: ocular features. *Bulletin de la Société Belge d'Ophtalmologie*.

[9] Herman J., Opitz J. M. (1969). A dominantly inherited first arch syndrome. *Birth Defects*.

[10] Braley A. E., Spivey B. E. (1963). Hereditary vitelline macular degeneration (possibly of vitelliform origin): a clinical and functional evaluation of a new pedigree with variable expressivity and dominant inheritance. *Transactions of the American Ophthalmological Society*.

